# Comment on: Impact of type 2 diabetes on complications after primary breast cancer surgery: Danish population-based cohort study

**DOI:** 10.1093/bjs/znae126

**Published:** 2024-05-27

**Authors:** Jatin Sridhar Naidu

**Affiliations:** Division of Surgery and Interventional Science, University College London, London, UK

*Dear Editor*

I extend my gratitude to Kjærgaard *et al.*^1^ for their recent contribution to breast surgery literature, a work of substantial interest to surgeons across various disciplines. Upon thorough review of their paper on type 2 diabetes mellitus (T2DM) and complications after primary breast cancer surgery, the authors admirably accounted for numerous covariates. However, their oversight in addressing factors like age, BMI and smoking status, known to independently heighten surgical complication risks, warrants attention. Despite acknowledging these limitations, it is worth noting that previous research has indicated that T2DM may not hold as significant a standalone risk factor when juxtaposed with variables like age or BMI, which are prevalent in T2DM patient cohorts. This raises concerns regarding potential overestimation of effect size within the study.

Although the authors conclude that the absence of T2DM in the presence of other co-morbidities suggests minimal impact on complication risks, such a conclusion may be misleading, given the nuances of the data set. Moreover, the study lacks essential information regarding the classification of the T2DM population, including diagnostic criteria and distinctions between prediabetic and diabetic statuses, understandably constrained by the Danish Breast Cancer Group (DBCG) database’s inherent limitations.

In view of these observations, it is imperative to interpret the study’s results with caution. Further studies are warranted to replicate findings while adequately controlling for confounding variables and effect modifiers. If reproducible, such findings could potentially inform the development of preventive strategies, such as antibiotic prophylaxis or enhanced glycaemic control preoperatively.
